# Mitochondrial Reactive Oxygen Species and Kidney Hypoxia in the Development of Diabetic Nephropathy

**DOI:** 10.3389/fphys.2017.00211

**Published:** 2017-04-11

**Authors:** Tomas A. Schiffer, Malou Friederich-Persson

**Affiliations:** ^1^Department of Medical Cell Biology, Uppsala UniversityUppsala, Sweden; ^2^Department of Medical and Health Sciences, Linköping UniversityLinköping, Sweden

**Keywords:** diabetic nephropathy, kidney hypoxia, mitochondrial function, superoxide production, mitochondrial uncoupling, mitochondrial ROS, hypoxia inducible factors

## Abstract

The underlying mechanisms in the development of diabetic nephropathy are currently unclear and likely consist of a series of dynamic events from the early to late stages of the disease. Diabetic nephropathy is currently without curative treatments and it is acknowledged that even the earliest clinical manifestation of nephropathy is preceded by an established morphological renal injury that is in turn preceded by functional and metabolic alterations. An early manifestation of the diabetic kidney is the development of kidney hypoxia that has been acknowledged as a common pathway to nephropathy. There have been reports of altered mitochondrial function in the diabetic kidney such as altered mitophagy, mitochondrial dynamics, uncoupling, and cellular signaling through hypoxia inducible factors and AMP-kinase. These factors are also likely to be intertwined in a complex manner. In this review, we discuss how these pathways are connected to mitochondrial production of reactive oxygen species (ROS) and how they may relate to the development of kidney hypoxia in diabetic nephropathy. From available literature, it is evident that early correction and/or prevention of mitochondrial dysfunction may be pivotal in the prevention and treatment of diabetic nephropathy.

## Diabetic nephropathy

Diabetic nephropathy accounts for ~45% of cases with end-stage renal disease (McCullough et al., 2007) and afflict about 30% of patients with diabetes mellitus (Hasslacher et al., 1989). Early diabetic nephropathy is evident as microalbuminuria (30–300 mg/day) and disease progression is characterized by progressively worsening albuminuria, loss of glomerular filtration rate, and structural changes such as thickening of glomerular basement membranes, extracellular matrix accumulation, and tubulointerstitial damage (Mauer et al., 1984; Brito et al., 1998; Katz et al., 2002; Mauer and Drummond, 2002). Currently, there are no curative treatments and disease progression ultimately results in requirement for renal replacement therapy i.e., dialysis or renal transplantation.

## Hypoxia is an acknowledged pathway to nephropathy

The oxygen levels in the kidney with oxygen tension in cortex around 50–60 mmHg and as low as 10–20 mmHg in medulla (Epstein et al., 1994) makes the kidney susceptible to diverse renal pathologies. The distribution of glycolytic enzymes has a heterogeneous pattern and are scarcely found from glomerulus until the loop of Henle with a multifold increase in thick ascending limb and along the rest of the nephron to the collecting duct (Guder and Ross, 1984). The proximal tubules therefore mainly rely on mitochondrial ATP production. However, the glycolytic enzymes in proximal tubules can be induced in hypoxia (Gullans et al., 1982; Dickman and Mandel, 1990). The theory of renal hypoxia has emerged as a pathway to nephropathy. It was suggested by Fine et al. that an initial glomerular injury would decrease blood flow through peritubular capillaries and decrease oxygenation, promoting tubulointerstitial fibrosis and damage progression, ultimately resulting in nephropathy (Fine et al., 1998). This theory is proposed to be a joint pathway for development of nephropathy in a number of conditions and not restricted to diabetic nephropathy alone. The chronic hypoxia theory has gained support in experimental animal studies (Palm et al., 2003; Ries et al., 2003; Rosenberger et al., 2008; Edlund et al., 2009; Haidara et al., 2009) as well as in human studies (Sayarlioglu et al., 2005; Hochman et al., 2007; Inoue et al., 2011). Navajo Indians living at high altitude have increased incidence of ESRD (non-diabetes-related; Hochman et al., 2007) and in patients suffering from sleep apnea the degree of nocturnal hypoxemia correlates with worsening kidney function (Sakaguchi et al., 2013). Also, patients with type 2 diabetes living at high altitude had increased incidence of diabetic nephropathy when compared to a similar patient group living at sea level. Importantly, glycaemia, hypertension, and lipidemia status were similar between the groups (Sayarlioglu et al., 2005). Expertly reviewed elsewhere, the support for kidney hypoxia as a common pathway to nephropathy is quite compelling (Nangaku, 2006; Singh et al., 2008; Mimura and Nangaku, 2010; Palm and Nordquist, 2011).

This review will not focus on the mechanisms of hypoxia resulting in nephropathy but rather the mechanisms that may influence the development of hypoxia, namely mitochondrial function and reactive oxygen species (ROS) production.

## Mitochondrial production of reactive oxygen species in the diabetic kidney

Production of ATP occurs in the mitochondrial inner membrane. In the electron transport system (ETS), the transferring of electrons from complex I to IV is coupled to translocation of protons to the intermembrane space, creating a membrane potential that is utilized by the ATP-synthase to produce ATP. Under normal conditions, ~0.1–0.2% of mitochondrial oxygen consumption is due to production of ROS (Kushnareva et al., 2002; St-Pierre et al., 2002). ROS production varies between different segments of the nephron where the medullary thick ascending limb of Henle (mTAL) is the predominant site of superoxide production in the kidney (Zou et al., 2001; Li et al., 2002). NADPH oxidase seems to be the main source of superoxide production in mTAL (Li et al., 2002) and tubular flow and luminal Na^+^ positively correlates with ROS production in this segment (Garvin and Hong, 2008; Cowley et al., 2015). Hall and colleagues demonstrated a higher total ROS production in proximal tubules compared to distal tubules (Hall et al., 2009). Inhibition of NADPH oxidase with apocynin revealed that the difference was attributed to higher NADPH oxidase activity in proximal tubules. The higher ratio of mitochondrial to nuclear volume in proximal tubules due to the larger cell size, indicate that the basal ROS production *per mitochondrion* is likely lower in proximal tubules. This fits with the lower membrane potential (Δψ_m_) and reduction grade of the electron transport chain in proximal tubules (Hall et al., 2009).

In the diabetic kidney, a large body of evidence supports the role ROS-induced damage (reviewed in Forbes et al., 2008) and oxidative markers such as 2-isoprostane, 8-hydroxy-2-deoxyguanosine, nitrotyrosine, and thiobarbituric acid reactive substances have been shown to increase in numerous studies with experimental models of diabetes and in diabetic patients (Broedbaek et al., 2011). In 2000, Nishikawa et al. put forward the view that mitochondrial superoxide was the source of oxidative stress in diabetes (Nishikawa et al., 2000). The reasoning was that cellular hyperglycemia would promote excessive pyruvate uptake into the mitochondria and therefore substrates feeding electrons to the ETS, ultimately resulting in hyperpolarization of the mitochondrial membrane and increased superoxide production. They demonstrated that hyperglycemia increased mitochondrial superoxide production that could be normalized by inhibiting complex II, uncoupling mitochondrial membrane potential by carbonyl cyanide m-chlorophenyl hydrazone or overexpression of uncoupling protein 1 (UCP-1) and by the addition of manganese superoxide dismutase (mnSOD). Importantly, the normalization of mitochondrial superoxide production prevented glucose-induced activation of known pathways to diabetes-induced damage: protein kinase C activation, nuclear factor kappa-light-chain-enhancer of activated B cells (NF-κB) activation, sorbitol accumulation, and the formation of advanced glycation end-products (Nishikawa et al., 2000).

Mitochondrial superoxide production is strongly regulated by mitochondrial membrane potential (Korshunov et al., 1997; Starkov and Fiskum, 2003; Lambert and Brand, 2004) and many reports show that mitochondria isolated from diabetic animals and cells cultured under hyperglycemic conditions display increased ROS production (Raza et al., 2004; Rosca et al., 2005; Yu et al., 2006; Quijano et al., 2007; Coughlan et al., 2009; Munusamy and MacMillan-Crow, 2009; Chacko et al., 2010; Sourris et al., 2012). Kidney cortex mitochondria isolated from type 2 diabetic db/db-mice show increased superoxide and hydrogen production that could be reduced by a mitochondrial antioxidant (Sourris et al., 2012). In kidney cortex of streptozotocin-induced diabetic rats, glycation of mitochondrial proteins was associated with decreased complex III-activity and increased superoxide production (Rosca et al., 2005). Coughlan et al. connected glucose-derived NADH (complex I substrate) to increased mitochondrial superoxide production in mesangial cells from diabetic rats (Coughlan et al., 2009). In bovine aortic endothelial cells, hyperglycemic culture conditions resulted in increased glucose metabolism, increased mitochondrial membrane potential, and increased formation of superoxide and hydrogen peroxide. Reducing mitochondrial membrane potential or inhibiting electron transport lowered mitochondrial ROS production whereas cells lacking mitochondria did not respond with increased ROS formation to hyperglycemic conditions (Quijano et al., 2007). Mitochondrial ROS production is also dependent on mitochondrial dynamics (Yu et al., 2006). Yu et al. showed in rat liver cells that upon high glucose exposure, mitochondria underwent rapid fragmentation and concomitantly increased ROS production. Inhibiting mitochondrial fragmentation prevented the glucose induced ROS production in several cell types (Yu et al., 2006, 2008).

Not all studies show increased mitochondrial ROS in diabetes. Real-time imaging and systemic administration of dihydroethidium (DHE) by Dugan et al. observed decreased superoxide production in intact kidneys of type 1 diabetic mice compared to control mice, also supported by electron paramagnetic resonance data in whole kidney homogenates. The reduced superoxide production was accompanied by hyper-phosphorylation of pyruvate dehydrogenase that contributes to deactivation of the enzyme. This leads to reduced conversion of pyruvate to acetyl coenzyme A and therefore reduced equivalents to the ETS that can reduce oxygen. The AMP-kinase (AMPK) activator 5-Aminoimidazole-4-carboxamide ribonucleotide (AICAR) restored the observed effects and the authors proposed a feed-forward cycle in which decreased AMPK activity would decrease mitochondrial biogenesis via peroxisome proliferator-activated receptor gamma coactivator 1-alpha (PGC-1α), a cycle that would be initiated and maintained through decreased mitochondrial ROS production (Dugan et al., 2013). However, DHE is prone to spontaneous oxidation and is affected by the general oxygen metabolism and technical aspects of its use are therefore important. Superoxide-specific DHE-metabolites can be separated by high performance liquid chromatography but not by its general fluorescence (Halliwell and Whiteman, 2004). Also, cortical tubular cells in diabetic animals have increased oxygen metabolism (Korner et al., 1994) with concomitantly decreased kidney oxygen tension (Palm et al., 2003) which will also affect general DHE-fluorescence even if the superoxide-specific products are still present. In the study by Dugan et al. measurements of ROS production were done in whole homogenate of kidneys (Dugan et al., 2013). Structural changes in diabetic nephropathy primarily affects the glomeruli and tubules, sites were diabetes-induced mitochondrial ROS-production has been reported. It may be postulated that measuring whole kidney ROS-production is not representative of alterations at the actual site of damage.

It has been proposed that the data in Dugan et al. (2013) can also be explained by glucose-induced increase in mitochondrial ROS production causing deoxyribonucleic acid (DNA) breaks in the nucleus, activating the repair enzyme poly adenosine diphosphate ribose polymerase (PARP). As NAD^+^ is a substrate for PARP its activation concomitantly reduces the NAD^+^ pool, reducing its availability for sirtuin 1, a known AMPK activator (Nishikawa et al., 2015). Therefore, PARP-activation could result in reduced AMPK activation creating a feed-forward loop of reduced AMPK and decreased mitochondrial biogenesis that is initiated by high mitochondrial ROS (Nishikawa et al., 2015). In contrast, Al-Kafaji et al. observed increased mitochondrial ROS with concomitant increased mitochondrial copy number in human mesangial cells as a result of high glucose exposure. These effects were abolished by addition of the MnSOD mimetic manganese (III) tetrakis (4-benzoic acid) porphyrin chloride (MnTBAP; Al-Kafaji and Golbahar, 2013).

It is important to remember that the inconclusive reports showing both higher and lower ROS production in the kidney in diabetes may represent a temporal effect in which the development of diabetic nephropathy may be a series of dynamic events where hyperglycemia may initially increase mitochondrial superoxide production but not able to maintain it as the disease progresses. Mitochondrial superoxide production may damage mitochondrial DNA and ETS itself, eventually resulting in reduced superoxide production (Xie et al., 2008; Tewari et al., 2012). Also, mitochondrial uncoupling, covered in the next section, may be an important mechanism to prevent excessive mitochondrial superoxide production.

## Mitochondrial uncoupling—a potentially detrimental consequence of increased ROS

Mitochondrial uncoupling is a process in which protons are released over the mitochondrial inner membrane independently of ATP-synthesis. This process results in the reduction of the mitochondrial membrane potential and therefore also decreased mitochondrial superoxide production (Nishikawa et al., 2000; Duval et al., 2002; Miwa and Brand, 2003; Starkov and Fiskum, 2003). However, in order to sustain ATP-production more electrons are transferred through the ETS and oxygen consumption is therefore increased. As the increased respiration is independent of ATP-production it is defined as leak-respiration and can be observed in isolated mitochondria under state 2 or 4 respirations or when the ATP-synthase is blocked by oligomycin. Physiologically, mitochondrial uncoupling is mediated by UCPs (Nicholls, 1976) that are sensitive to inhibition by purine nucleotides (Jaburek et al., 1999; Echtay et al., 2001) but can also be mediated by the adenine nucleotide transporter that is sensitive to carboxyatractyloside (Shabalina et al., 2006). UCP-2 is expressed in human kidney mitochondria (Fleury et al., 1997) as well as in rats and mice (Jezek et al., 1999; Friederich et al., 2009). In kidney cortex from type 1 diabetic rats UCP-2 expression was increased, resulting in increased mitochondrial leak-respiration that was sensitive to purine nucleotides (Friederich et al., 2008).

UCPs are proposed as the mitochondrial internal defense system against increased ROS production and several reports show that UCPs can be activated by superoxide (Echtay et al., 2002; Krauss et al., 2003) as well as by lipid peroxidation products (Echtay et al., 2003). In support, kidney cortex mitochondria of type 2 diabetic db/db-mice displayed increased mitochondrial leak respiration that was corrected after treatment with the antioxidant Q10 (Persson et al., 2012). The role of UCP-2 in regulating mitochondrial membrane potential and therefore ROS production has been reported in several studies. In murine endothelial cells, small interfering (si)-RNA toward UCP-2 resulted in increased mitochondrial membrane potential and ROS production (Duval et al., 2002). Macrophages from UCP-2 knockout mice display increased ROS production (Arsenijevic et al., 2000) and these mice display increased survival and clearance rates following infections (Arsenijevic et al., 2000; Rousset et al., 2006). In mice, the overexpression of UCP-2 in the brain is reported to decrease lesions and enhance neurological functions after ischemic insults (Mattiasson et al., 2003). Interestingly, after siRNA-mediated silencing of UCP-2 in diabetic rats it was observed that the adenine nucleotide transporter compensated with a further increased mitochondrial uncoupling in kidney cortex mitochondria (Friederich-Persson et al., 2012), indicating mitochondrial uncoupling as a mechanism of importance in the diabetic kidney.

While some report increased mitochondrial membrane potential with diabetes or hyperglycemic culture conditions (Nishikawa et al., 2000; Raza et al., 2004; Rosca et al., 2005; Yu et al., 2006; Quijano et al., 2007; Coughlan et al., 2009; Munusamy and MacMillan-Crow, 2009; Chacko et al., 2010; Sourris et al., 2012) not all studies do (Friederich-Persson et al., 2012; Persson et al., 2012). Studies that did not observe hyperpolarized mitochondria in diabetic kidneys also show the presence of mitochondrial uncoupling and increased mitochondrial membrane potential was only observed when mitochondrial uncoupling was blocked (Friederich-Persson et al., 2012; Persson et al., 2012). Thus, mitochondrial uncoupling may be a defensive strategy in order to maintain mitochondrial membrane potential at a normal level. Temporal effects may also be important. Munusamy et al. cultured proximal tubular cells under hyperglycemic condition and saw an initial increase in mitochondrial membrane potential and increased ROS-production. Importantly, this was followed by a second phase with reduced mitochondrial membrane potential and ROS production (Munusamy and MacMillan-Crow, 2009). It may be postulated that the second phase represents the initiation of mitochondrial uncoupling but the use of purine nucleotides or carboxyatractyloside were not employed to probe for possible mechanisms of leak respiration (Munusamy and MacMillan-Crow, 2009).

While mitochondrial uncoupling can be viewed as a mechanism protective of mitochondrial function it may be detrimental for the kidney due to the resulting increased oxygen consumption. The kidney is unable to correct increased oxygen usage with increased renal blood flow since that would increase tubular load of electrolytes destined for active transport and therefore in itself increase oxygen demand. Therefore, increased mitochondrial leak respiration would cause a decrease in renal oxygen availability and it has been suggested that diabetes-induced mitochondrial uncoupling contributes to the low oxygen tension seen in diabetic kidneys (Friederich-Persson et al., 2012). Indeed, treating healthy rats with the mitochondrial uncoupler dinitrophenol for 30 days results in kidney hypoxia, albuminuria, and tubulointerstitial damage without affecting levels of glycaemia or oxidative stress (Friederich-Persson et al., 2013).

## AMPK signaling in the diabetic kidney

AMPK regulates anabolic processes and the AMPK activity is dependent upon the surrounding energy levels (Hardie et al., 2012). When AMP/ATP ratio is increased under conditions such as hypoxia, exercise and hypoglycemia AMPK is allosterically activated (Hardie et al., 2012). In addition, AMPK can also be activated by upstream kinases such as the liver kinase B1 (Hawley et al., 2003) and Ca^2+^/calmodulin-dependent protein kinase kinases β (Emerling et al., 2009). When activated, AMPK contributes to the inhibition of anabolic energy-consuming pathways and stimulates catabolic energy-producing pathways, thus leading to increased intracellular energy levels (Hardie et al., 2003).

In the diabetic kidney, despite renal hypoxia, AMPK is downregulated (Dugan et al., 2013). One possibility is that the degree of hypoxia may not be severe enough to result in AMPK-activation. Another possibility is the role of mitochondrial ROS production. Nishikawa recently proposed a hypothesis where ROS-induced activation of PARP may reduce the levels of NAD^+^ and thus AMPK-activity through reduced SIRT1-activity (Nishikawa et al., 2015). Also, Emerling and colleagues reported that hypoxic activation of AMPK is dependent on mitochondrial ROS production rather than AMP:ATP ratios (Emerling et al., 2009). ROS also appear to mediate calcium release from the endoplasmic reticulum which then contributes to the formation of CRAC channels. This contributes to amplification of the signal through the entry of calcium that subsequently activates Ca^2+^/calmodulin-dependent protein kinase kinases β that targets AMPK (Mungai et al., 2011). On the contrary, Dugan et al. observed decreased levels of ROS in the diabetic kidney and suggested a feed-forward cycle of decreased AMPK, PGC-1α, and mitochondrial biogenesis initiated and maintained by reduced mitochondrial ROS production (Dugan et al., 2013).

AMPK has emerged as a regulator of cellular redox state and expression of antioxidants through the class O forkhead box signaling pathway (Sanchez et al., 2012) that promotes cell survival, mitochondrial biogenesis, and longevity (Fernandez-Marcos and Auwerx, 2011; Martins et al., 2016). In response to metabolic stress, class O forkhead box 3 is activated by AMPK that subsequently leads to increased levels of endogenous antioxidants such as mnSOD as well as thioredoxin, peroxiredoxin, and catalase (Kops et al., 2002; Zrelli et al., 2011). Therefore, AMPK activation may have the potential to attenuate glucose-induced oxidative stress in diabetic nephropathy. The antidiabetic and AMPK activating drug metformin lowered ROS production, lipid peroxidation and increased the antioxidant system in kidneys in a rat model of gentamicin toxicity (Morales et al., 2010). HUVEC cells treated with AMPK activator AICAR displayed decreased hyperglycemia-induced ROS production, increased expression of PGC-1α, mnSOD, nuclear respiratory factor-1, and mitochondrial transcription factor A together with stimulated mitochondrial proliferation (Kukidome et al., 2006).

**Figure 1:**
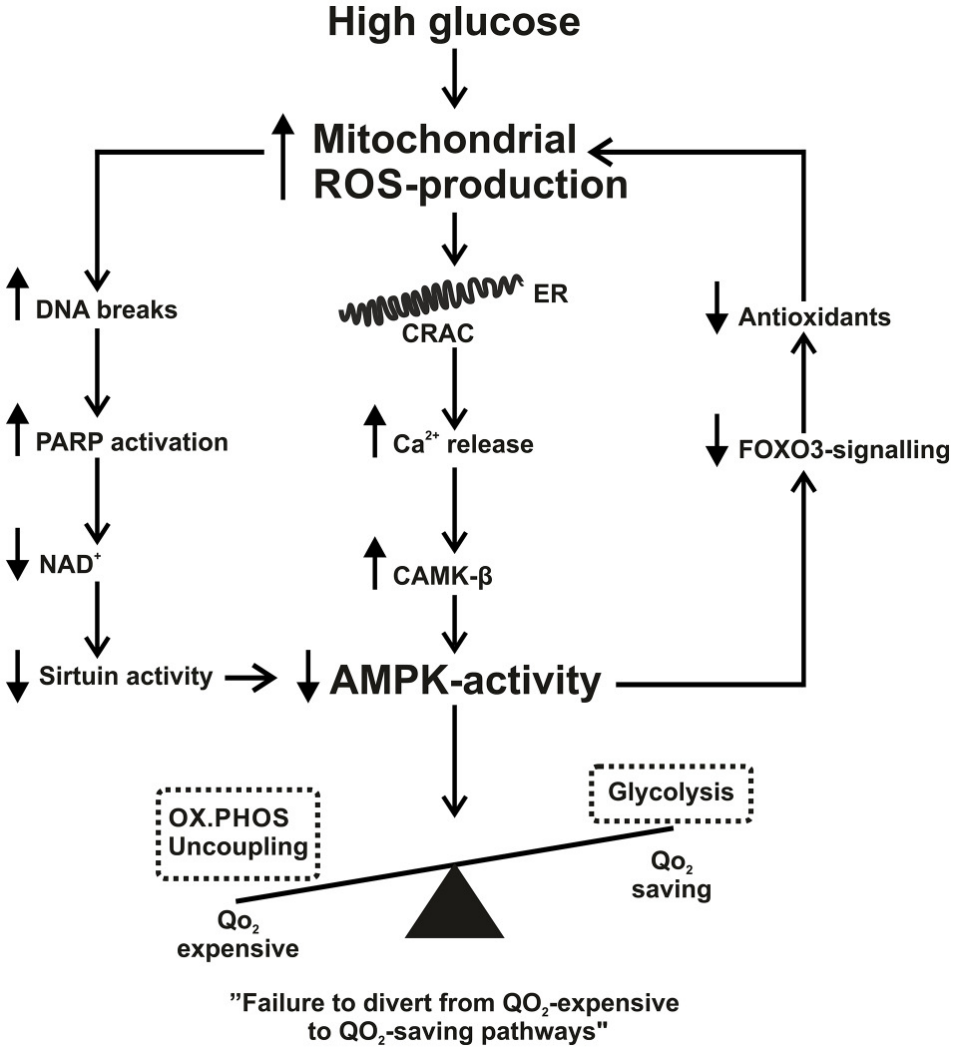
**Summary scheme of how increased mitochondrial reactive oxygen species production (ROS) connects to decreased 5′ AMP-activated protein kinase activity**. See text for further details. AMPK, AMP-activated protein kinase; CAMK-β, Ca^2+^/calmodulin-dependent protein kinase; CRAC, calcium release activated channel; ER, endoplasmic reticulum; FOXO3, Forkhead box O3; NAD, nicotinamide adenine dinucleotide; OX.PHOS, oxidative phosphorylation; PARP, poly (ADP-ribose) polymerase; ROS, reactive oxygen species; QO_2_, oxygen consumption.

The conflicting literature regarding mitochondrial ROS production in diabetes makes the relationship between mitochondrial ROS production and AMPK activation especially difficult to interpret. However, if mitochondrial ROS production is increased with hyperglycemia, locally if not represented in the whole kidney, not only would increased mitochondrial ROS production result in mitochondrial uncoupling and contribute to hypoxia through that pathway but also result in reduced AMPK activation and thus fails to divert oxygen consumption from oxidative phosphorylation to the more oxygen-saving glycolysis. As AMPK is reported to be down-regulated in the diabetic kidney (Dugan et al., 2013), AMPK activators may have beneficial effects on kidney hypoxia. In cultured liver epithelial cells, metformin suppressed hypoxia inducible factor (HIF)-1α stabilization by decreasing cellular oxygen consumption (Minokoshi et al., 2004; Zhou et al., 2016). No studies to date have investigated whether AMPK-activators can affect kidney oxygenation *in vivo*. See Figure 1 for summary of proposed mechanisms.

Some caution is needed regarding possible off target effects of AMPK activators. Inhibition of hypothalamic AMPK is necessary for leptin's negative effects on food intake and body weight (Minokoshi et al., 2004; Hardie, 2015) and pharmacological AMPK activation may therefore alter the hormonal and nutrient-derived signals and energy balance (Hardie, 2015). Achieving tissue specific AMPK activation would therefore be optimal in any future AMPK-dependent treatments.

## Hypoxia inducible factors in the diabetic kidney

The oxygen dependent degradation of HIF-1α regulates the activity of HIF-1 transcription factor complex (Chen and Sang, 2016). In normoxia, prolyl 4-hydroxylases hydroxylate HIF-1α, enables the binding to the von-Hippel-Lindau complex and subsequent proteosomal degradation by an E3 ubiquitin ligase complex (Chen and Sang, 2016). The stabilization or degradation of HIF is a swiftly regulated process. Upon reoxygenation after hypoxia, degradation of the HIF-1α protein occurs with a half-life of <1 min (Yu et al., 1998). Recent studies highlight that ROS partly play a role in the stabilization of HIF-1α (Irwin et al., 2009; Niecknig et al., 2012; Zepeda et al., 2013). Mitochondrial ROS in particular (Brunelle et al., 2005) and sirtuin-dependent transactivation of HIF have emerged as a key factor (Lim et al., 2010; Zhong et al., 2010; Finley et al., 2011; Hubbi et al., 2013). HIF-target genes include pyruvate dehydrogenase kinase 1 (Loenarz et al., 2011) that inhibits pyruvate dehydrogenase, reducing the entry of acetyl-coenzyme A to the tricarboxylic acid cycle (TCA) and therefore contributes to the hypoxia-induced switch from oxidative phosphorylation to glycolysis. Sustained HIF-signaling also induces angiogenesis through vascular endothelial growth factor (Conway et al., 2001) and hematopoiesis via erythropoietin (Semenza, 1999) and fibrotic encapsulation will occur in long-term hypoxia (Tanaka, 2017).

Systemic hypoxia leads to accumulation of HIF-1α in most tubular segments with varied intensity, depending on the nature of hypoxic stimulus (Rosenberger et al., 2002). By contrast, HIF-2α is expressed in endothelial cells of a small subset of glomeruli, peritubular endothelial cells and fibroblasts (Rosenberger et al., 2002). Interestingly, inducing HIF by the hypoxia mimetic cobalt chloride mainly contributes to HIF-1α in distal convoluted tubuli presumably related to uptake and accumulation (Nagao et al., 1999). Induced HIF-1α expression in the kidney has been demonstrated with diabetes in both rats (Rosenberger et al., 2008) and humans (Higgins et al., 2007; Shao et al., 2016) where overall HIF-signaling may contribute to renal injury. Higgins et al. showed that genetic ablation of epithelial HIF-1α in primary renal epithelial cells and in proximal tubules of kidneys subjected to unilateral ureteral obstruction inhibited the development of tubulointerstitial fibrosis, decreased inflammatory cell infiltration and reduced the number of fibroblast-specific protein-1-expressing interstitial cells (Higgins et al., 2007). Renal HIF-1α expression is also associated with tubulointerstitial injury in patients with chronic kidney disease (Higgins et al., 2007) and increased area of renal fibrosis has been observed in tubular epithelial cell specific von-Hippel-Lindau knockout mice (Kimura et al., 2008). Silencing HIF-1α with short hairpin RNA-technique significantly attenuated levels of collagen and α-smooth muscle actin and injury in kidneys from hypertensive rats (Wang et al., 2014). In addition, Nayak and colleagues observed reduced whole kidney glomerular hypertrophy, mesangial matrix expansion, extracellular matrix accumulation and urinary albumin excretion in diabetic mice upon treatment with the HIF-1 inhibitor YC-1 (Nayak et al., 2016).

In contrast, Nordquist et al. found no increase in HIF-responsive genes measured in whole kidney homogenates after 4 weeks of streptozotocin-induced diabetes where renal hypoxia was present (Nordquist et al., 2015). In this study diabetes-induced renal hypoxia, proteinuria and kidney injury were prevented by chronic HIF-activation via cobalt chloride (Nordquist et al., 2015), highlighting the importance of HIF-activation on regulating oxygen metabolism in diabetic nephropathy (further highlighted in Haase, 2015). Chronic HIF-activation has also been reported to be beneficial in an obese hypertensive model of type 2 diabetes where cobalt chloride did not affect metabolic parameters, obesity or hypertension but reduced proteinuria, improved kidney histology and decreased expression of fibrotic markers transforming growth factor β and connective tissue growth factor (Ohtomo et al., 2008).

Interestingly, kidney cortex mitochondria from diabetic animals in Nordquist et al. displayed mitochondrial uncoupling that was completely prevented by HIF-activation (Nordquist et al., 2015). In pulmonary artery smooth muscle cells, deficiency of mitochondrial UCP-2 resulted in hyperpolarized mitochondria, resistance to apoptosis, and reduced TCA cycle intermediates, changes that were replicated by hypoxia in wild type pulmonary artery smooth muscle cells. These results were substantiated in UCP-2 knockout mice when the mice displayed a pseudohypoxic state with increased pulmonary HIF-1α signaling, vascular remodeling and spontaneous development of pulmonary hypertension (Dromparis et al., 2013). It has been shown that mitochondrial ROS contributes to the stabilization of HIF (Brunelle et al., 2005), this through a ROS mediated activation of p38 mitogen-activated protein kinase (Emerling et al., 2005). However, as mitochondrial uncoupling proteins are also activated by ROS (Echtay et al., 2002) and some studies report normalized membrane potential in kidney cortex mitochondria isolated from diabetic rats and mice (Friederich-Persson et al., 2012; Persson et al., 2012) the glucose-induced mitochondrial uncoupling may act to prevent HIF-activation. Rosenberger et al. also observe this in a study where HIF-signaling could be enhanced by Tempol administration (Rosenberger et al., 2008), further suggesting an involvement of ROS. It has also been suggested that hyperglycemia directly interferes with HIF-signaling through hyperosmolarity as shown in endothelial cells and dermal fibroblasts (Catrina et al., 2004). Together, mitochondrial ROS and hyperglycemia may act to render the HIF-activation in diabetic kidneys submaximal.

Summarizing available data, a case can be made both for and against using chronic HIF-1α activation in diabetic nephropathy. Disparate results may well reflect the use of different models of diabetes but is also likely to reflect temporal aspects of HIF-activation; how long should HIF-activation be sustained and early or late in disease progression? These are just some of the issues that should be clarified in further studies.

## Mitochondrial dynamics and mitophagy in the diabetic kidney

In healthy tissues, mitochondria are present in tubular networks that are constantly reworked by mitochondrial fission and fusion. Fission results in mitochondrial fragmentation and production of short rods and spheres whereas fusion results in long filamentous mitochondria. It is a highly dynamic process (Liesa et al., 2009; Westermann, 2010) and controlled by a set of proteins where dynamin related protein 1 (DRP1) and mitochondrial fission 1 protein (FIS1) controls fission and mitofusin 1–2 and optic atrophy 1 is in control of fusion (Chan, 2006).

As previously mentioned, mitochondrial dynamics is important for glucose-induced increase in mitochondrial ROS production (Yu et al., 2006). Wang et al. elegantly showed the role of Rho-associated coiled coil-containing protein kinase 1 in activation and recruitment of DRP1 to mitochondria upon high glucose stimulation, promoting mitochondrial fission, fragmentation, and higher ROS production in podocytes (Wang et al., 2012). High glucose exposure, expression of DRP1 and FIS1 is induced in endothelial cells together with a loss of the mitochondrial network (Shenouda et al., 2011). Inhibiting DRP1 can also attenuate acute kidney injury and tubular cell apoptosis in mice (Brooks et al., 2009). Similarly, boosting mitochondrial fusion by overexpressing mitofusion 2 resulted in reduced proteinuria, kidney hypertrophy and albumin:creatinine ratio together with improved pathological changes in diabetic rats (Pawlikowska et al., 2007). Mitochondrial fission and concomitantly increased ROS production that contributes to the deleterious impact on kidney function is evident in diabetes. Thus, inhibiting fission and/or promoting fusion may impede the progression of DN.

Selective degradation of mitochondria by the autophagic machinery is termed mitophagy (Lemasters, 2005) and mitochondria must undergo fragmentation into spheroids before being encapsulated within autophagic vesicles (Twig et al., 2008). Dysfunctional mitochondria that have lost their membrane potential are tagged for mitophagy clearance via the PTEN-induced putative kinase (Matsuda et al., 2010; Vives-Bauza et al., 2010), a serine/threonine-protein kinase located on the mitochondrial outer membrane. PTEN-induced putative kinase is a docking protein for parkin, a key protein to direct damaged mitochondria for engulfment by autophagosomes (Matsuda et al., 2010; Narendra et al., 2010; Vives-Bauza et al., 2010; Lazarou et al., 2013).

The observed accumulation of damaged mitochondria in the kidney in diabetes indicates impairment in the mitophagy system (Kimura et al., 2011; Liu et al., 2012; Takahashi et al., 2012) and autophagy has previously been linked to the pathogenesis of diabetic nephropathy, acute kidney injury and polycystic kidney disease (Huber et al., 2012). Sequestosome 1 (P62/SQSTM1) is an autophagy marker that correlates inversely to autophagic flux (Vallon et al., 2013). In a rat type 2 diabetes model, p62/SQSTM1 was increased (Kitada et al., 2011) and reduced levels of autophagy markers were observed in mouse glomerular lysates 28 days after streptozotocin injection (Fang et al., 2013). Decreased staining of the autophagy marker, LC3-phosphatidylethanolamine conjugate (LC3-II) was also observed in podocytes from human biopsies obtained from patients with diabetes (Fang et al., 2013). Thioredoxin interacting protein, another mitophagy regulator, is upregulated and results in increased ROS production, inflammation and fibrosis in the diabetic kidney (Devi et al., 2012; Mahmood et al., 2013).

Cellular homeostasis is dependent on a dynamic balance between fission and fusion and it is clear that in mitochondrial dynamics and mitophagy is affected in the diabetic kidneys. Many reports relate this to mitochondrial ROS production that will in turn affect pathways related to kidney hypoxia such as AMPK-signaling, HIF-activity and mitochondrial uncoupling. Interestingly, Galloway and colleagues showed that inhibiting mitochondrial fission in hepatocytes overexpressing dynamin like protein (DLP-1) caused increased proton leak with a functionally intact electron transport chain (Galloway et al., 2012). Whether pharmacological blockade of mitochondrial fission is beneficial in diabetic nephropathy is currently unclear and it should be cautioned that chronic disruption of fission might eventually induce mitochondrial dysfunction via uncoupling, accumulation of damaged mitochondria, and cell injury.

## Summary and conclusions

The development of diabetic nephropathy is likely to represent a series of dynamic events. Many studies report altered mitochondrial function in the diabetic kidney. These alterations include altered mitophagy, mitochondrial dynamics, mitochondrial uncoupling, and signaling through AMPK and HIF. Herein, we have discussed how these pathways may relate to mitochondrial ROS production in the diabetic kidney. In experimental models, correcting mitochondrial ROS production reduces albuminuria in diabetic mice (Sourris et al., 2012) and preventing ROS induced mitochondrial leak-respiration improves kidney function (Persson et al., 2012). However, a recent phase 3 study where the nuclear respiratory factor 2-inducer bardoxolone methyl was used for targeting diabetic nephropathy failed to improve the outcome of end-stage renal disease or cardiovascular mortality. Instead, increased albuminuria, heart-failure, and hypertension in patients with advanced diabetic nephropathy contributed to early termination of the study (de Zeeuw et al., 2013).

**Figure 2:**
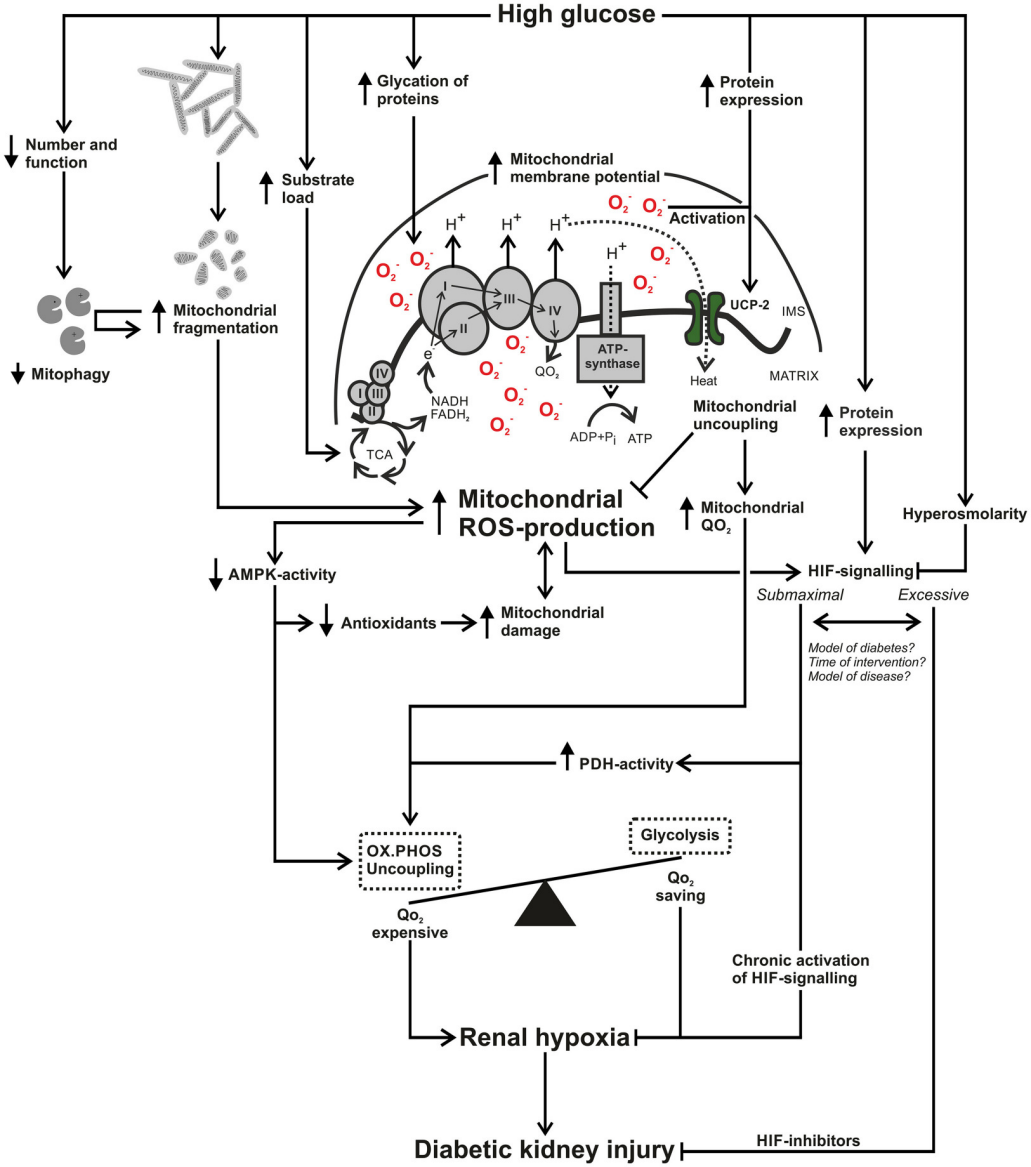
**Schematic summary of how increased mitochondrial reactive oxygen species (ROS) production connects to renal hypoxia and diabetic kidney injury**. High glucose results in increased mitochondrial ROS-production through glycation and damage of electron transporting complexes and an increased load of electron donating substrates, resulting in an increased mitochondrial membrane potential. High glucose results in mitochondrial fragmentation and due to glucose-induced alterations in mitophagy there may be an accumulation of damaged and fragmented kidney mitochondria in diabetes. Mitochondrial ROS-production can reduce activation of 5′ AMP-activated protein kinase (AMPK), resulting in decreased antioxidant systems, creating a circle that contributes to mitochondrial damage and perhaps further enhanced ROS-production. Decreased AMPK-activity will also fail to divert oxygen consumption (QO_2_)-expensive pathways such as oxidative phosphorylation to QO_2_-saving pathways such as glycolysis. High glucose and increased mitochondrial ROS production will increase expression and activation of uncoupling protein-2. The process of mitochondrial uncoupling will reduce mitochondrial membrane potential and ROS production but will concomitantly increase mitochondrial QO_2_. High glucose can increase hypoxia inducible factor (HIF)-1α expression and mitochondrial ROS-production contributes to HIF activation. Hyperosmolarity can interfere with HIF signaling and chronic activation of HIF can prevent renal hypoxia. On the other hand, various inhibitors of HIF attenuates renal injury, raising the issue whether kidney HIF signaling is submaximal or excessive in diabetes. Differences in study results may involve methodological setup in terms of model of diabetes or other kidney disease but also the time point of intervention. In summary, increased mitochondrial ROS-production affects pathways in manners that can contribute to increased kidney QO_2_ and may therefore be an important mechanism in the development of kidney hypoxia and diabetic kidney injury. AMPK, AMP-activated protein kinase; FAD, flavin adenine dinucleotide; H^+^, proton; HIF, hypoxia inducible factor; IMS, intermembrane space; NAD, nicotinamide adenine dinucleotide; ${\text{O}}_{2}^{.-}$, superoxide ion; OX.PHOS, oxidative phosphorylation; PDH, pyruvate dehydrogenase; TCA, tricarboxylic acid cycle; ROS, reactive oxygen species; UCP, uncoupling protein; QO_2_, oxygen consumption.

Many of the discussed factors are intertwined in a complex manner, making it very difficult to highlight a specific mechanism in the diabetic kidney. Partly underlying the difficulty of highlighting a specific mechanism is the functional heterogeneity of the kidney. Different segments have different function and surrounding oxygen levels, different levels of the discussed cellular pathways and different sources and levels of ROS production. However, from available literature it seems clear that mitochondrial ROS production may be a joint mechanism. The resulting effects on mitochondrial and cellular pathways affecting oxygen metabolism may play an important role in the development of diabetic nephropathy. For a schematic summary, see Figure 2. The correction and/or prevention of mitochondrial dysfunction may be pivotal in the prevention and treatment of diabetic nephropathy.

## Author contributions

TS and MFP both wrote manuscript, viewed the content critically and approved the final submitted version.

## Funding

MFP is supported by the Wenner-Gren Foundations.

### Conflict of interest statement

The authors declare that the research was conducted in the absence of any commercial or financial relationships that could be construed as a potential conflict of interest.

## Abbreviations

AICAR: 5-Aminoimidazole-4-carboxamide ribonucleotide
AMPK: AMP-kinase
DHE: dihydroethidium
DNA: deoxyribonucleic acid
DRP: dynamin related protein
DLP: dynamin like protein
ETS: electron transport system
FIS: mitochondrial fission protein
HIF: hypoxia inducible factor
LC3-II: LC3-phosphatidylethanolamine conjugate
NF-κB: nuclear factor kappa-light-chain-enhancer of activated B cells
mnSOD: manganese superoxide dismutase
MnTBAP: manganese (III) tetrakis (4-benzoic acid) porphyrin chloride
P62/SQSTM1: sequestosome 1
PARP: poly adenosine diphosphate ribose polymerase
PGC-1α: peroxisome proliferator-activated receptor gamma coactivator 1α
ROS: reactive oxygen species
Si: small interference
TCA: tricarboxylic acid
UCP: uncoupling protein.
